# The MicroRNA-148/152 Family: Multi-faceted Players

**DOI:** 10.1186/1476-4598-12-43

**Published:** 2013-05-19

**Authors:** Yue Chen, Yong-Xi Song, Zhen-Ning Wang

**Affiliations:** 1 Department of Surgical Oncology and General Surgery, First Hospital of China Medical University, Shenyang 110001, P. R. China; 2 Department of Colorectal Surgery, Liao Ning Cancer Hospital & Institute, Shenyang 110042, P. R. China

**Keywords:** miR-148/152 family, Disease, Normal tissue

## Abstract

MicroRNAs(miRNA) are noncoding RNAs of about 19–23 nucleotides that are crucial for many biological processes. Members of the microRNA-148/152(miR-148/152) family, which include microRNA-148a(miR-148a), microRNA-148b(miR-148b), and microRNA-152(miR-152), are expressed differently in tumor and nontumor tissues and are involved in the genesis and development of disease. Furthermore, members of the miR-148/152 family are important in the growth and development of normal tissues. Members of the miR-148/152 family regulate target genes and are regulated by methylation of CPG islands. In this review, we report recent studies on the expression of members of the miR-148/152 family, methylation of CPG islands, and their target genes in different diseases, as well as in normal tissues.

## MicroRNA biogenesis

MiRNAs are noncoding RNAs of about 19–23 nucleotides. They are transcribed by RNA polymerase II into pri-miRNAs. These are processed by RNAse III Drosha into 70 to 100-nucleotide pre-miRNAs [1]. Pre-miRNAs, mediated by the RNAse III Dicer, generate double-stranded RNAs approximately 22 nucleotides long [2]. These are miRNAs/miRNAs*, which are mature miRNA guides and miRNA* complementary passenger strands. One of the two strands is selected as a guide strand based on thermodynamic properties; the complementary miRNA* strand is usually degraded [3]. Moreover, miRNAs are posttranscriptional regulators that bind by complementary base-pairing to sequences in the 3′-Untranslated Regions(3′-UTR)of target mRNAs, resulting in downregulation [4]. Growing evidence indicates that more and more miRNAs play key roles in a wide variety of biological processes including cell fate specification, proliferation, cell death, and energy metabolism through altering the expression of targets by both downregulation [5] and upregulation [6].

## Structure of the miR-148/152 family

**Figure 1:**
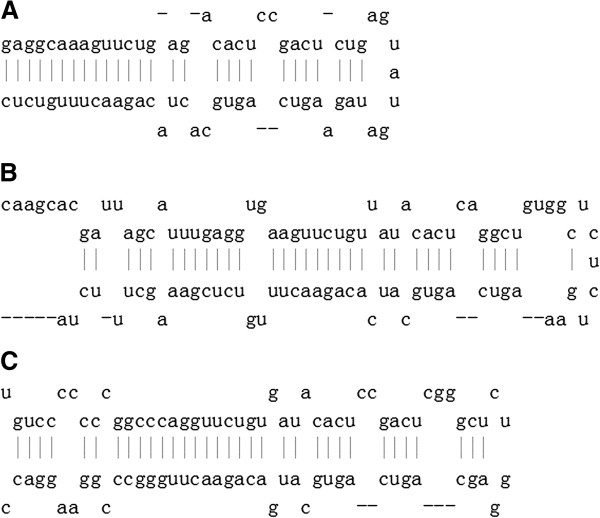
**Stem-loop structure of pre-miR-148/152 family.** (**A**) Pre-miR-148a. (**B**) Pre-miR-148b. (**C**) pre-miR-152.

**Figure 2:**
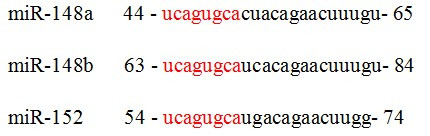
Mature sequence of miR-148/152 family (The same seed sequence are marked by red font).

MiR-148a, miR-148b, and miR-152 are the three members of the miR-148/152 family [7]. The pre-miR-148/152 family members have a stem-loop structure (Figure 1) that is processed into the mature members of the miR-148/152 family by a series of intranuclear and intracytoplasmic enzymes. Mature members of the miR-148/152 family are 21–22 nucleotides in length, with the same seed sequence of approximately 6–7 nucleotides (Figure 2). The seed sequence is an important region for binding to target mRNAs. MiR-148/152 family members are involved in various biological processes through complementary binding between the seed sequence and the 3′-UTR of target mRNAs. Numerous tumors and normal tissues express the miR-148/152 family members differently during growth, development, and tumorigenesis. Therefore, miR-148/152 family members might be critical for these processes.

## Functions of miR-148/152 family members in normal tissue

MiR-148/152 family members have aberrant expression in normal tissue, especially in stem cells. Merkerova et al. investigated miR-148a expression in hematopoietic stem cells (HSCs) and found that miR-148a was decreased in HSCs [8]. MiR-148a was also downregulated in mesenchymal stem cells compared to embryonic stem cells and in osteodifferentiated multipotent mesenchymal stromal cells compared to multipotent mesenchymal stromal cells [9,10]. Schoolmeesters et al. reported that miR-148b was upregulated in the osteogenesis of early osteogenic differentiation of human mesenchymal stem cells [11]. Zhang et al. identified miR-148a as a novel myogenic miRNA that mediated myogenic differentiation. Expression levels of miR-148a increased during C2C12 myoblast differentiation [12]. In a study of mouse adipogenesis, John et al. found that miR-148b expression was increased [13]. In a study of hepatic injury and rejection after liver transplantation, Farid et al. found that miR-148a expression was significantly reduced in liver tissue with prolonged graft warm ischemia times. Conversely, serum levels were elevated in patients with liver injury and this positively correlated with aminotransferase levels. These findings might provide early, sensitive and specific biomarkers of liver injury [14]. In a study of dendritic cells, which are important in linking the innate and adaptive immune responses, Liu et al. demonstrated that miR-148b/miR-152 family members were negative regulators of the innate response and the antigen-presenting capacity of dendritic cells by targeting CaMKIIalpha. This function might contribute to immune homeostasis and immune regulation [15]. Manaster et al. showed that in placental tissue, both miR-148a and miR-152 were expressed at relatively low levels compared with other healthy tissues. In placental tissue, levels of human leukocyte antigen G (HLA-G), a miR-148a and miR-152 target gene, were high and therefore important for a healthy pregnancy [16].

Expression of miR-148/152 family members might be altered by exposure to certain physical and chemical factors. Palmieri et al. identified 16 upregulated and 2 downregulated miRNAs in osteoblast-like cells line (MG-63) cultured with Medpor, an alloplastic material used for craniofacial reconstruction. In these conditions, the expression of miR-148b and miR-152 were upregulated. These results might provide a better understanding of the molecular mechanism of bone regeneration and a model for comparing other materials with similar clinical effects [17]. Wang et al. studied miRNA expression profiles in brains of fetal mice with prenatal ethanol exposure. MiR-152 was one of the upregulated miRNAs [18]. Wu et al. demonstrated that miR-148b was upregulated 1.53-fold in response to radiation treatment in non-Hodgkin’s lymphoma (NHL). Further research in Raji cells indicated that miR-148b sensitized Raji cells to radiotherapy. These results demonstrated that miR-148b increased the radiosensitivity of Raji cells and suggested that miR-148b was important in the response of NHL to ionizing radiation [19] Wang et al. determined miRNA expression profiles in the human lung fibroblasts cell line WI-38 exposed to ionizing radiation (IR). They identified four upregulated miRNAs including miR-152. These results suggested that miRNAs are involved in the regulation of IR-induced senescence. Therefore, targeting these miRNAs might be a novel approach for modulating the cellular response to radiation exposure [20].

## MiR-148/152 family and disease: upregulation and downregulation

Aberrant expression of MiR-148/152 family has been observed in tumor [7,21] and nontumor [22] diseases. Many studies have identified that MiR-148/152 family members potentially acted as oncogenes and tumor suppressors. Moreover, the growing evidence has demonstrated that miR-148/152 family members also played important roles in some nontumor diseases, such as IgA nephropathy [22], type 1 diabetes [23], atherosclerotic lesions [24], chronic fatigue syndrome/myalgic encephalomyelitis [25].

### Upregulation of miR-148/152 family

MiR-148/152 family members are upregulated in many diseases. Huang et al. reported that six miRNAs including miR-148a were significantly upregulated in the plasma of multiple myeloma (MM) and high levels of miR-148a were related to shorter relapse-free survival times [21]. Also in plasma, Cuk et al. noted that miR-148b was significantly upregulated in breast cancer patients [26]. Moreover, Yuan et al. reported that miR-148a was upregulated in hepatitis B cells associated with hepatocellular carcinoma (HCC) [27]. Furthermore, Gokhale et al. found miR-148a, as one of abnormal expressed miRNAs, was overexpressed in the WNT signaling-associated medulloblastomas [28]. Therefore, miR-148a and miR-148b might be significant biomarkers in these cancer patients and might provide an early easy detection method.

In IgA nephropathy, miR-148b, which potentially targets core 1 synthase, glycoprotein-N-acetylgalactosamine 3-beta-galactosyltransferase 1 (C1GALT1), was upregulated [22]. Nielsen et al. found 12 upregulated human miRNAs, including miR-152, in the serum of type 1 diabetes patients relative to age-matched healthy controls [23]. Moreover, monocytes are critical in atherosclerotic lesion formation, and can be subdivided into classical and nonclassical subsets [29]. Bidzhekov et al. studied miRNA expression profiles of atherosclerotic plaques and found that miR-99b and miR-152 were co-expressed in plaque tissue and classical monocytes [24]. Taken together, these findings increase our understanding of the importance of miR-148/152 family in nontumor diseases.

### Downregulation of miR-148/152 family

MiR-148/152 family members are decreased in various tumor types, indicating that they have the potential to act as tumor-suppressor miRNAs. Li et al. found miR-148b was underexpressed in liver cancer stem cells (LCSCs) [30]. In a study of hepatic cell lines, Zhao et al. found that miR-148b was downregulated in the liver cancer cell lines HepG2, MHCC97L, and MHCC97H relative to the hepatic cell line L02 [31]. Moreover, Huang et al. reported that miR-152 was downregulated in HBV-related HCC tissues compared with adjacent noncancerous hepatic tissues [32]. In view of the above, we speculated that miR-148/152 family members were downregulated in hepatocellular carcinoma. Furthermore, in hepatoblastoma, the expression of miR-148a was demonstrated to be lower than that in hepatocellular carcinoma [33].

In gastrointestinal cancers, Chen et al. noted that miR-148a and miR-152 were downregulated in cancer tissue and cancer cell lines [7]. Furthermore, they also found that low expression of miR-148a and miR-152 correlated with increased tumor size and advanced pT stage [7]. Moreover, the study of Zheng et al. revealed that the low expression of miR-148a was significantly associated with lymph node metastasis in gastric cancer [5]. They further found that Rho-associated, coiled-coil containing protein kinase 1(ROCK1), which might be a target of miR-148a, was involved in miR-148a-induced suppression of gastric cancer cell migration and invasion [5]. Especially, the studies of Song et al. showed that miR-148b was downregulated in gastric cancer [34], colorectal cancer [35] and suppressed cell growth by targeting cholecystokinin-2 receptor(CCK2R). These results highlighted that miR-148/152 family might play important roles in gastric cancer progression and would become a potential biomarker.

In a study of cholangiocarcinoma, DNA methyltransferase 1(DNMT1) was verified as a target for miR-148a and miR-152 and the expression level of these miRNAs was decreased in cancer cells [36]. In pancreatic ductal adenocarcinoma, Liffers et al. reported that miR-148a exhibited significant downregulation compared with normal pancreatic ductal cells and further investigation proved that miR-148a regulated cell survival through targeting cell division cycle 25B(CDC25B) [37]. Moreover, in an animal model of oral squamous cell carcinoma, Yu et al. observed that expression of miR-148b was downregulated among 12 miRNAs [38].

Furthermore, the expression of miR-148/152 family members is low not only in digestive system, but also in genital system tumors. Zhou et al. observed that the expression of miR-152 was decreased in ovarian cancer tissue and ovarian cancer cell lines, but miR-148a expression was decreased only in cancer cell lines [39]. Hiroki et al. noted that reduced miR-152 expression correlated significantly with poor overall survival and disease-free survival in endometrial serous adenocarcinomas [40]. On the other hand, MiR-148a was also downregulated in hormone-refractory prostate cancer cells (PC3 and DU145) and overexpression of miR-148a could inhibite cell growth, cell migration, invasion by targeting Mitogen- and stress-activated kinase 1 (MSK1) [41]. In cancer-associated fibroblasts, Aprelikova et al. showed that miR-148a was downregulated compared with matched normal tissue fibroblasts established from patients with endometrial cancer and wingless-type MMTV integration site family, member 10B (WNT10B) was a direct target of miR-148a [42]. In some nontumor diseases, such as chronic fatigue syndrome/myalgic encephalomyelitis (CFS/ME), miR-152 was significantly decreased in NK cells of CFS/ME patients compared with nonfatigued controls [25].

In summary, studies of miR-148/152 family members showed that their expression levels decreased in HCC, LCSC, gastrointestinal cancers, cholangiocarcinoma, pancreatic ductal adenocarcinoma, oral squamous cell carcinoma, ovarian cancer, endometrial serous adenocarcinoma and prostate cancer. MiR-148/152 family members might be tumor-suppressive miRNAs in these tumors.

## MiR-148/152 family and methylation

DNA methylation of miR-148/152 family member genes was found in many tumors types. Interaction has been observed between DNA methylation and miR-148/152 family members through one of their target genes: DNMT1. Zhu et al. demonstrated that, in gastric cancer, miR-148a was inactivated by hypermethylation of DNA in the promoter region of its gene; this was mediated through DNMT1 overexpression. Silencing of miR-148a reduces its suppression of DNMT1 in gastric cancer, and this might result in overexpression of DNMT1, promoting DNA hypermethylation [43]. Hanoun et al. found that hypermethylation of the DNA region encoding miR-148a was responsible for its low expression in pancreatic ductal adenocarcinoma samples and in preneoplastic pancreatic intraepithelial neoplasia lesions [44]. Lujambio et al. used a pharmacological approach with a DNA demethylating agent to show that miR-148a, miR-34b/c, and miR-9 underwent specific hypermethylation-associated silencing in cancer cells compared with normal tissues. Most important, they found that DNA methylation-associated silencing of tumor suppressor miRNAs might contribute to the development of human cancer metastasis [45]. Stumpel et al. identified 11 miRNAs, including miR-152, that were downregulated in t(4;11)-positive infant acute lymphoblastic leukemiaas a consequence of CpG hypermethylation. Futher study showed that both myeloid/lymphoid or mixed-lineage leukemia(MLL) and DNMT1 were potential targeted genes of miR-152 and the high degree of methylation of the miR-152 CpG island was strongly correlated with poor clinical outcome [46]. Based on these two studies, we hypothesize that methylation of CpG islands in miR-148/152 family member genes might induce particular biological behaviors of cancer. Pavicic et al. studied inherited, familial carcinoma, including colorectal, gastric and endometrial carcinomas, and found increased DNA methylation of miR-148a and 152 in tumor tissues compared with normal tissues. In particular, hypermethylation at miR-148a and miR-152 genes was associated with microsatellite-unstable tumors. This study highlighted the importance of epigenetic DNA methylation of miRNA genes in hereditary cancers [47]. In another breast cancer study, Xu et al. found that DNMT1 was overexpressed and this overexpression was responsible for hypermethylation of miR-148a and miR-152 promoters. As an miR-148a/152 target, DNMT1 was inversely related to the expression levels of miR-148a/152. This study revealed that a novel miR-148a/152-DNMT1 regulatory circuit might exist in breast cancer [48].

Therefore, methylation of miR-148/152 family member genes might occur at CpG islands, reducing expression of miR-148/152 family members. Expression of DNMT1, which is an important gene for DNA methylation and is a target gene of miR-148/152 family members, is inversely restricted to the expression level of miR-148a/152. This might result in overexpression of DNMT1, promoting DNA methylation. A novel miR-148a/152-DNMT1 regulatory circuit might exist in tumors.

## MiR-148/152 family members and target genes

In different cellular contexts, one miRNA perhaps can regulate diverse pathways and cause various phenotypes depending on the availability of a certain population of mRNA targets [49]. MiR-148/152 family members have many different targets and whether they are important to function depends on their specific target mRNAs [36,50]. MiRNA targets are predicted mainly by three computational algorithms: TargetScan [51], PicTar [52] and miRBase targets [53]. Moreover, microRNA arrays, real-time PCR, luciferase reporter assays and western blots are the main methods for investigating miRNA targets. To validate the targets of miR-148/152 family members, the following two criteria must be met: firstly, the expression of their target gene correlates inversely with miR-148/152 family members. Secondly, they have miR-148/152 family members binding sites with complementary sequences, which can directly bind to miR-148/152 family members. Luciferase reporter assay would be needed. The luciferase constructs with a target 3′UTR are specifically responsive to miR-148/152 family members. Inversely, deletion or mutation of the miR-148/152 family members binding sites from the 3′UTR abolishes the miR-148/152 family members regulation [49]. The targets are summarized in Table 1.

**Table 1:** Targets of miR-148/152 family in various tissues/cells

**miR-148/152 family**	**Expression**	**Biological function**	**Targets**	**Tissues/cells**	**References**
miR-148a			PXR	Human liver	[54]
miR-148a	Upregulation	DNA hypomethylation	DNMT1	CD4+ T cells of Systemic lupus erythematosus	[55]
miR-148a		Tumor growth	CAND1	Human prostate cancer	[50]
miR-148a		Apoptosis	Bcl-2	Colorectal cancer	[56]
miR-148a	Downregualtion	Cell proliferation	p27	Gastric cancer	[57]
miR-148a			HLA-C	HIV-1 infected individuals	[58]
miR-148a			ACVR1	HeLa cell	[59]
miR-148a, miR-152	Downregualtion	Immune rejection	HLA-G	Placenta tissue	[16]
miR-148a	Downregualtion		WNT10B	Cancer-associated fibroblasts	[42]
miR-148a		Cell proliferation,cycle progression,migration	PTEN	Hepatocellular carcinoma	[27]
miR-148a		Cellgrowth, migration,invasion	MSK1	Prostate cancer cells	[41]
miR-148a	Downregualtion	Cell growth	CDC25B	Human pancreatic ductal adenocarcinoma	[37]
miR-148a	Downregulation	Cell invasion and metastasis	ROCK1	Gastric cancer	[5]
miR-148b	Downregulation	Cell proliferation	CCKBR	Gastric cancer	[34]
miR-148b	Downregulation	Cell proliferation	CCK2R	Colorectal cancer	[35]
miR-148a, miR-152	Downregulation		CCKBR?*	Gastrointestinal cancer	[7]
miR-148a, miR-152	Downregualtion	Cell proliferation	DNMT-1	Malignant cholangiocytes	[36]
miR-148b	Upregulation	Glycosylation of IgA1	C1GALT1	IgA nephropathy	[22]
miR-148a, miR-152	Downregualtion	Cell proliferation, colony formation, tumor angiogenesis	IGF-IR, IRS1	Breast cancer	[48]
miR-148a, miR-152	Downregualtion		DNMT1	HBV-related hepatocellular carcinoma	[32]
miR-152			DNMT1?, MLL?	Acute lymphoblastic leukemia	[46]
miR-152		NK cell-mediated cytolysis	HLA-G?	Human trophoblast cell line (JEG-3)	[60]
miR-148/152 family			CaMKIIalpha	TLR-triggered dendritic cells	[15]
miR-152		Cell motility and adhesion	CSF-1	Ovarian cancer cell	[61]
miR-152		Cell growth	DNMT1, Rictor	Endometrial cancer	[62]

*Question mark presents target gene to be further validated.

DNMT1, which is a DNA methyltransferase enzyme, mediate the transfer of methyl groups from S-adenosylmethionine to the 5 position of cytosine bases in the dinucleotide sequence CpG [63]. DNMT1 is important in tumorigenesis. Studies have shown that DNMT1 is abnormally expressed in many tumor types [64,65], and their regulation by miR-148/152 family members has been reported in a number of human diseases including systemic lupus erythematosus [55], cholangiocarcinoma [36], hepatocellular carcinoma [32], acute lymphoblastic leukemia [46] and endometrial cancer [62]. These might indicate that miR-148/152 and DNMT1 would be a significant pair in the induction and progression of human diseases. Furthermore, an interaction between DNMT1 and miR-148a/152 was found in breast cancer. This study revealed that a novel miR-148a/152-DNMT1 regulatory circuit might exist in breast cancer [48].

PTEN is a phosphatase that catalyzes the conversion of the lipid second messenger PtdIns(3,4,5)*P*3 to phosphatidylinositol (4,5)-bisphosphate [PtdIns(4,5)*P*2] [66]. PTEN mutations occur frequently in a variety of human cancers, such as endometrial carcinoma [67], glioblastoma multiforme [68], skin [69] and prostate cancers [70]. PTEN, as a target gene, has been reported to be regulated by a variety of miRNAs, such as miRNA-21 [71], miRNA-22 [72] and miRNA-26a [73]. In miR-148/152 family members, only miR-148a was reported [27]. Further investigation would be needed.

CCKBR, also called CCK2R, has proliferative effects on various cancer, such as gastric, colorectal, pancreatic and small cell lung cancer through gastrin [74-77]. It is confirmed as a target gene of miR-148b by our research group in recent years [34,35]. The follow-up studies are proceeding in our group.

Genes such as PXR, CAND1, HLA-C, ACVR1, IGF-IR and IRS1 represent targets of miR-148/152 family members and changes in the expression of these miRNAs are associated with cell motility or(and) cell growth. PXR is a major transcription factor regulating the inducible expression of a variety of transporters and drug-metabolizing enzymes. Takagi et al. revealed that miR-148a could recognize the miR-148a recognition sequence of PXR mRNA by reporter assay. The PXR protein level was decreased by the overexpression of miR-148a, whereas it was increased by inhibition of miR-148a in human liver [54]. Murata et al. determined that miR-148a reduced the expression of CAND1 by binding to the 3′-UTR of CAND1 mRNA and promoted the growth of human prostate cancer [50]. In the study of HLA-C, which associated with HIV, Kulkarni et al. confirmed HLA-C was a target gene of miR-148a. ACVR1, which was correlated with endothelial-to-mesenchymal transition in endothelial cells, was verified a target gene of miR-148a [59]. In breast cancer, Xu et al. revealed that miR-148a and miR-152 acted as tumor suppressors by targeting IGF-IR and IRS1, which mediate key mechanisms of tumor growth and progression [48]. Furthermore, Bcl-2 [56], p27 [57], CSF-1 [61] and Rictor [62] were also demonstrated to be targets of miR-148/152 family members.

## Conclusions

Members of the miR-148/152 family including miR-148a, miR-148b and miR-152, have been found to have different roles in various tissues such as tumor, nontumor and normal tissues. Whether upregulated or downregulated in tissues, the miR-148/152 family is involved in regulating target genes, such as genes for proliferation, differentiation and apoptosis. MiR-148/152 family members are regulated by methylation of their CpG islands. A novel miR-148a/152-DNMT1 regulatory circuit might exist. In conclusion, although great progress has been made in recent years, the molecular mechanisms of miR-148/152 family members and their function in different tissues remain unclear and should be investigated in future studies.

## Abbreviations

miRNA: microRNAs; miR-148/152: microRNA-148/152; miR-148a: microRNA-148a; miR-148b: microRNA-148b; miR-152: microRNA-152; 3′-UTR: 3′-Untranslated Regions; HSCs: Hematopoietic stem cells; HLA-G: Human leukocyte antigen G; NHL: Non-Hodgkin’s lymphoma; IR: Ionizing radiation; MM: Multiple myeloma; HCC: Hepatocellular carcinoma; C1GALT1: Core 1 synthase, glycoprotein-N-acetylgalactosamine 3-beta-galactosyltransferase 1; LCSCs: Liver cancer stem cells; ROCK1: Rho-associated, coiled-coil containing protein kinase 1; CCK2R: Cholecystokinin-2 receptor; DNMT1: DNA methyltransferase 1; CDC25B: Cell division cycle 25B; MSK1: Antigen identified by monoclonal antibody AJ9; WNT10B: Wingless-type MMTV integration site family, member 10B; CFS/ME: Chronic fatigue syndrome/myalgic encephalomyelitis; MLL: Myeloid/lymphoid or mixed-lineage leukemia; PXR: Pregnane X receptor; CAND1: Cullin-associated and neddylation-dissociated 1; Bcl-2: B-cell CLL/lymphoma 2; HLA-C: Human leukocyte antigen C; ACVR1: Activin A receptor, type 1; PTEN: Phosphatase and tensin homolog; CDC25B: Cell division cycle 25B; CCKBR: Cholecystokinin B receptor; IGF-IR: Insulin-like growth factor-I receptor; IRS1: Insulin receptor substrate 1; CaMKIIalpha: Calcium/calmodulin-dependent protein kinase II alpha; CSF-1: Colony stimulating factor-1; Rictor: RPTOR independent companion of MTOR, complex 2.

## Competing interests

The authors declare that they have no competing interests.

## Authors’ contributions

YC collected and read the related paper and drafted the manuscript. YXS cooperated and helped to draft the manuscript. ZNW participated in the design of the review and helped to draft the manuscript. All authors read and approved the final manuscript.
